# RNA-Seq Reveals Adaptation Strategy in Grass Carp (*Ctenopharyngodon idella*) Under Hypersaline Conditions

**DOI:** 10.3390/ijms26072930

**Published:** 2025-03-24

**Authors:** Tao Zhu, Hongmei Song, Zhu Zhu, Jing Tian, Caixia Lei, Jinxing Du, Shengjie Li

**Affiliations:** Key Laboratory of Tropical and Subtropical Fishery Resource Application and Cultivation, Ministry of Agriculture and Rural Affairs, Pearl River Fisheries Research Institute, Chinese Academy of Fishery Sciences, Guangzhou 510380, China; zhut1009@163.com (T.Z.); shm@prfri.ac.cn (H.S.); 13940846346@163.com (Z.Z.); zjtianjing@prfri.ac.cn (J.T.); leicaixia0703@sina.com (C.L.); m18621151103@163.com (J.D.)

**Keywords:** grass carp, salt tolerance, aquaculture, transcriptome

## Abstract

Grass carp (*Ctenopharyngodon idella*) is a key aquaculture species, and understanding its adaptation mechanisms to saline environments is crucial for addressing the global freshwater salinization challenge. In this study, juvenile grass carp were acclimated to three salinity levels (0, 4, and 8 ppt) for 30 days, after which gill and intestinal tissues were sampled to quantify cortisol concentrations and conduct RNA-seq. Results showed that cortisol levels exhibited a salinity-dependent increase, with significantly higher concentrations in gill tissues than in intestinal tissues, suggesting that cortisol plays an important role in the salt adaptation of grass carp. RNA-seq revealed that ion transport-related genes were upregulated in gills, whereas biosynthesis, oxygen transport, and energy metabolism genes were downregulated. In the intestine, genes involved in taurine transport and intercellular junctions were highly expressed, while immune-related genes showed reduced expression. These findings suggest that high salinity suppresses respiration and energy metabolism efficiency, with ion exchange primarily occurring in gills. Functional annotation identified seven candidate genes (*LOC127513882*, *aqp9b*, *ca4a*, *ca5a*, *igfbp1b*, *slc12a2*, and *slc12a4*) as key regulators of salinity adaptation. Overall, our study provides valuable insights into the mechanisms underlying the salt tolerance of grass carp.

## 1. Introduction

Under the combined pressures of climate change, industrial pollution, and human activities, global freshwater salinization is becoming increasingly severe. In natural ecosystems, salinization poses significant threats to the health of freshwater environments, resulting in heightened rates of aquatic deformities, increased mortality, and loss of biodiversity [1,2]. At the same time, freshwater salinization has a profound impact on aquaculture. It not only affects growth, leading to decreased growth rates and increased energy expenditure [3,4], but also alters immune responses, increasing the risk of infection. Particularly in inappropriate salinity levels, the activity of immune enzymes in aquaculture organisms can be inhibited, rendering them more vulnerable to pathogen invasion [5,6]. Consequently, investigating the mechanisms of salt tolerance in fish is essential for promoting the sustainable development of aquaculture.

The grass carp (*Ctenopharyngodon idellus*) is naturally distributed in China, Russia, and Bulgaria. In China, it is primarily found in the Yangtze River and Pearl River systems, as well as associated lakes, reservoirs, and ponds [7]. It is highly favored by aquaculture producers due to its well-established breeding technology, low input costs, high survival rates, and stable consumer demand [8]. Currently, the grass carp has become the most significant freshwater aquaculture species in China, with a production volume of 5.94 million tons in 2024, accounting for 17.4% of the total freshwater aquaculture output [9]. Furthermore, grass carp has been introduced to many regions, including Asia, Europe, the Americas, and Africa [10]. In China, it is cultivated in all 30 provinces. The main production areas are concentrated in the Central South, East China, and Southwest regions. However, in the northwestern and northeastern regions of China, there are extensive high-salinity water bodies with salinity levels reaching 6‰ or higher. Due to their excessive salinity, these water bodies are generally unsuitable for agricultural production or conventional aquaculture. Promoting saline aquaculture of grass carp in these areas holds significant importance, as it not only optimizes the utilization of water resources but also stimulates local economic development.

The effects of freshwater salinization on grass carp are complex. Research indicates that grass carp can adapt effectively to maintain stable growth and physiological conditions in low-salinity environments [4]. However, exposure to high-salinity environments can inhibit growth and induce physiological and immune alterations, resulting in reduced disease resistance and increased susceptibility to infections [6]. At the molecular level, several key proteins have been identified as crucial regulators of salt tolerance in fish. Among these, proteins involved in ion transmembrane transport, such as NKCC, Na^+^/K^+^-ATPase, and CFTR, play a direct role in the transport of ions across cellular membranes [11,12]. These proteins are primarily responsible for the transmembrane transport of sodium, chloride, and potassium ions. When freshwater fish are exposed to high-salinity environments, the expression levels of these proteins significantly increase, facilitating the maintenance of osmotic pressure balance. This adaptive response is essential for ensuring cellular homeostasis and overall physiological stability in saline conditions. Additionally, certain hormones, including thyroid hormone, growth hormone, and insulin-like growth factor, have been found to indirectly regulate salt tolerance in fish [13,14]. The advent of transcriptome sequencing technology has led to the discovery of numerous genes associated with salt tolerance in fish. Extensive research has revealed that fish exhibit differential expression of genes related to ion transport, energy metabolism, immunity, and tight junction proteins in response to high salinity conditions [15,16,17]. Notably, differentially expressed genes in fish salt tolerance studies vary significantly across species, indicating species-specific differences in salt tolerance-related genes.

To investigate the mechanisms underlying the adaptation of grass carp to high-salinity environments, identify key genes regulating salinity adaptation processes, and provide a theoretical foundation for breeding new high-salt-tolerant grass carp varieties. This study raised grass carp in three different salinity levels (0, 4, and 6 ppt) for 30 days. The intestines and gills were collected to measure cortisol concentrations and perform RNA-seq. Comparative transcriptome analysis was employed to investigate the osmoregulatory adaptation mechanism of grass carp.

## 2. Results

### 2.1. The Cortisol Concentration in Gills and Intestine

**Figure 1:**
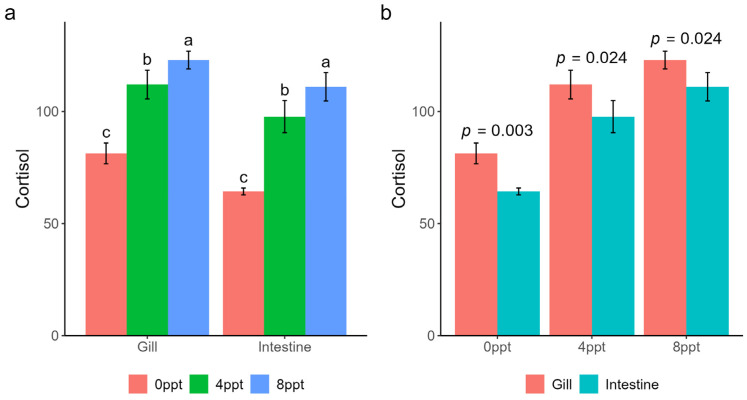
Cortisol concentrations in the gills and intestines: (**a**) grouped by organ, the letters on the bar chart represent the significance of multiple comparisons; (**b**) grouped by salinity, the numbers denote the *t*-test *p*-values.

Cortisol levels were quantified in 24 samples across all experimental groups, with 4 biological replicates per group. The results of cortisol concentration detection showed that with the increase in salinity, cortisol levels in the gills increased from 81.3 ± 4.6 to 112.0 ± 6.4, while cortisol levels in the intestine increased from 64.3 ± 1.5 to 111.0 ± 6.3 (Figure 1a). Moreover, across the three different salinities, the cortisol concentration in the gills was higher than that in the intestine (*p* < 0.05) (Figure 1b).

### 2.2. Transcriptome Sequencing and Gene Expression Analysis

**Figure 2:**
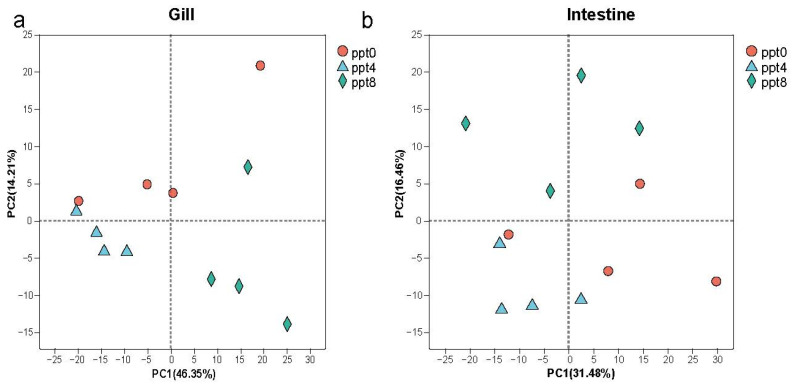
Principal component analysis of gene expression levels. (**a**). Principal component analysis of gills. (**b**). Principal component analysis of intestinal.

In this study, a total of 24 samples, comprising 2 tissues, 3 groups, and 4 replicates, were sequenced, yielding 163.2 Gb of clean data. Each sample produced over 6.0 Gb of clean data, with a Q30 base percentage exceeding 95.26% (Table S1). Following quality control, the clean reads for each sample were mapped to the grass carp reference genome, achieving alignment rates between 90.17% and 94.88% (Table S1), which indicates excellent sequencing quality and high representativeness of the expression levels. After transcript assembly, read counting, and TPM conversion, we generated an expression matrix containing 31,777 genes. Principal component analysis showed significant aggregation of samples from different groups in both gills and intestines (Figure 2a,b).

### 2.3. Analysis of Gill Gene Expression Patterns

**Figure 3:**
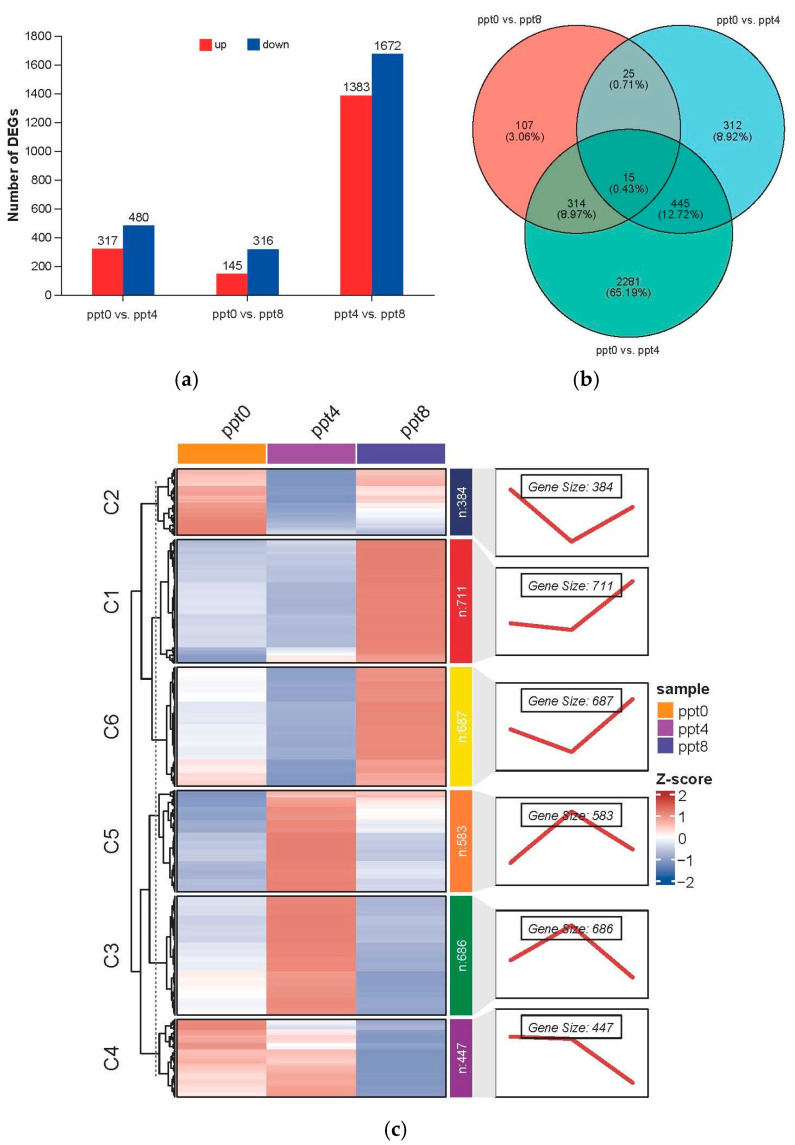
Analysis of differentially expressed genes in gills. (**a**) The number of upregulated and downregulated genes in the three comparisons. (**b**) Venn plots of DEGs across the three comparisons. (**c**) In cluster analysis of DEGs, the colors in the heatmap represent expression levels, the numbers on the right side of the heatmap indicate the number of genes, and the line chart illustrates the trend of gene expression.

To obtain differentially expressed genes (DEGs), three comparisons were established: ppt0 vs. ppt4, ppt0 vs. ppt8, and ppt4 vs. ppt8. Among these three comparisons, the ppt4 vs. ppt8 comparison exhibited the highest number of DEGs, totaling 3055, while the other two comparisons had 797 and 461 DEGs, respectively (Figure 3a and Table S2). By comparing the intersections of DEGs, a total of 3499 genes were found to be differentially expressed in at least one comparison, with 15 genes differentially expressed across all three comparisons (Figure 3b). Among these 15 genes, the expression level of LOC127501982 increased with rising salinity but has not been functionally characterized. The remaining genes exhibited u-shaped or n-shaped expression patterns, among which two genes (slc16a4 and slc27a1b) were associated with transmembrane substance transport.

DEGs showing significant changes in at least one comparison were subjected to trend analysis, with the optimal cluster number determined as K = 6 based on a minimal within-cluster sum of squares (Figure S1). Among the six clusters, Cluster 2 (384 genes) and Cluster 6 (687 genes) exhibited u-shaped expression profiles, while Cluster 3 (686 genes) and Cluster 5 (583 genes) displayed n-shaped expression profiles; Cluster 1 (711 genes) maintained stable expression at 0–4 ppt but showed dramatic upregulation at 8 ppt, inversely correlated with Cluster 4 (447 genes) exhibiting marked downregulation at 8 ppt (Figure 3c).

**Figure 4:**
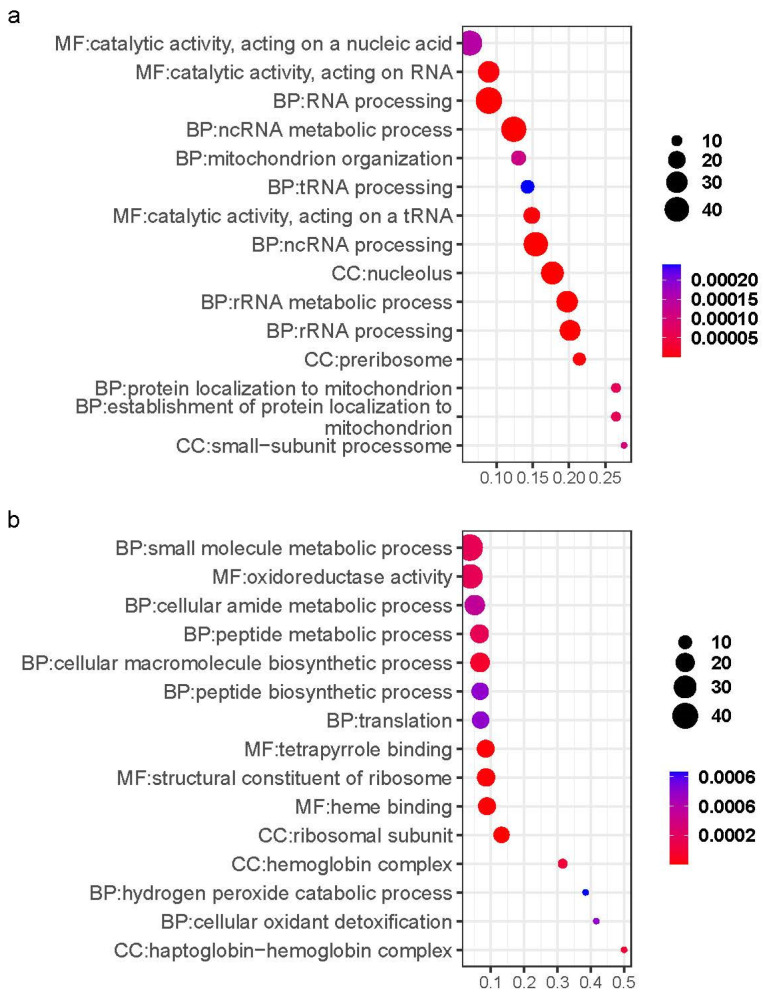
The 15 most significant GO terms for Cluster 1 (**a**) and Cluster 4 (**b**).

Functional enrichment of Cluster 1 genes identified 771 significant GO terms (FDR < 0.05), where the top 15 pathways were enriched in RNA-related processes (Figure 4a), with 9.8% (76/771) linked to ionic transport mechanisms, including GO:0015294 (solute/cation symporter activity), GO:0015370 (solute/sodium symporter activity), and GO:0006814 (sodium ion transport). Key sodium/potassium/chloride transporters included LOC127513882 (sodium/potassium ATPase α1 subunit), slc12a2, and slc13a5a, alongside proton transport genes atp6v1aa and LOC127507303 (slc45a3 homolog) associated with GO:0046961 (proton-transporting ATPase activity) and GO:0009678 (pyrophosphate-driven proton transport).

Functional enrichment analysis of Cluster 4 with downregulated expression identified 466 significant GO terms, where the top 15 pathways were predominantly associated with small molecule metabolism, oxygen-hemoglobin transport, biosynthesis, and ATP metabolic processes, while the remaining terms similarly converged on these functional categories (Figure 4b). Small molecule metabolism/biosynthesis-related genes primarily involved ribosomal protein family members, including rplp0, rps12, rps15, and rps21, oxygen transport gene involved hemoglobin subunits such as LOC127509166 (hemoglobin cathodic β-like subunit), LOC127509173 (hemoglobin cathodic α-like subunit), hbaa2, hbba1, and hbba2, energy metabolism-associated genes comprised LOC127525128 (kynurenine 3-monooxygenase), LOC127525483 (cytochrome P450 7A1 homolog), ehhadh, gpd1b, sirt2, and sirt3.

### 2.4. Analysis of Intestinal Gene Expression Patterns

**Figure 5:**
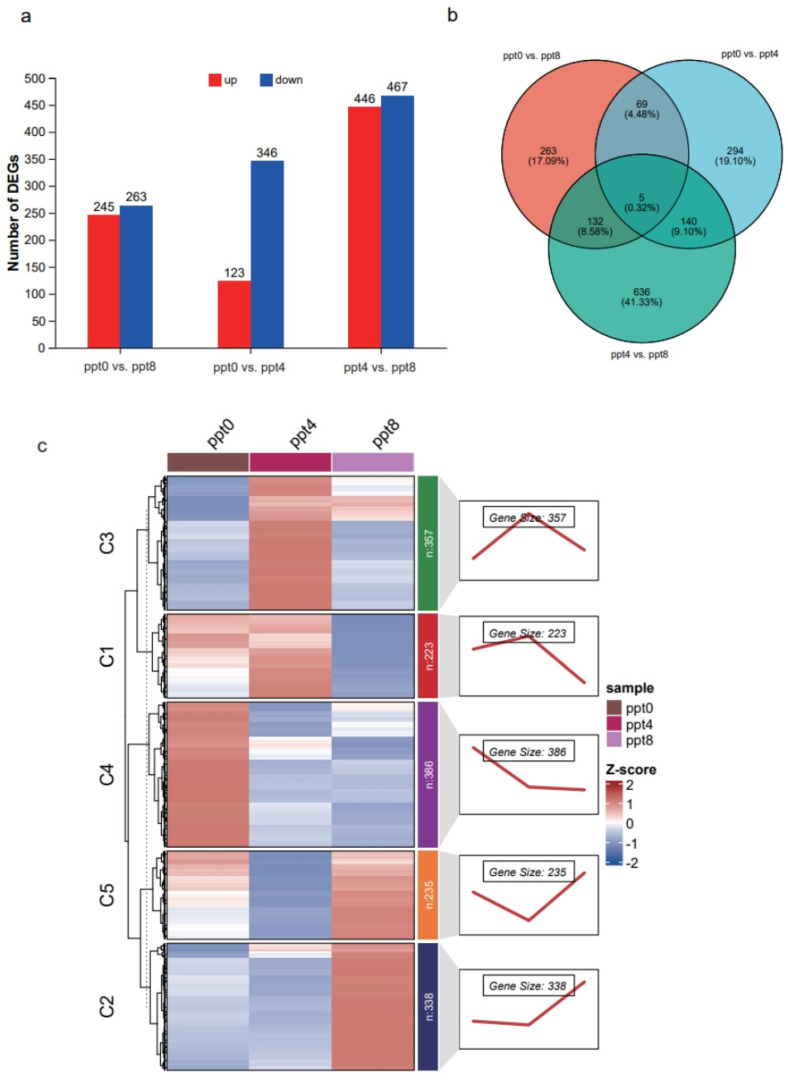
Analysis of differentially expressed genes in the intestine. (**a**) The number of upregulated and downregulated genes in the three comparisons. (**b**) Venn plots of DEGs across the three comparisons. (**c**) In cluster analysis of DEGs, the colors in the heatmap represent expression levels, the numbers on the right side of the heatmap indicate the number of genes, and the line chart illustrates the trend of gene expression.

In the intestine, pairwise comparisons between salinity groups identified 469 (0 vs. 4 ppt), 508 (0 vs. 8 ppt), and 913 (4 vs. 8 ppt) DEGs, with the 4 vs. 8 ppt comparison showing the highest DEG count (Figure 5a, Table S3). After deduplication, 1539 non-redundant genes showed differential expression in at least one comparison, with only 5 genes shared across all three companions(Figure 5b). Cluster analysis of all DEGs determined K = 5 as the optimal cluster number (Figure S2), partitioning genes into clusters 1–5 containing 223, 338, 357, 386, and 235 genes, respectively, with expression patterns illustrated in Figure 5c. Cluster 2 exhibited stable expression at 0–4 ppt but showed dramatic upregulation at 8 ppt, whereas Cluster 4 displayed the opposite trend. Clusters 1, 3, and 5 displayed u-shaped or n-shaped expression patterns as salinity increased.

**Figure 6:**
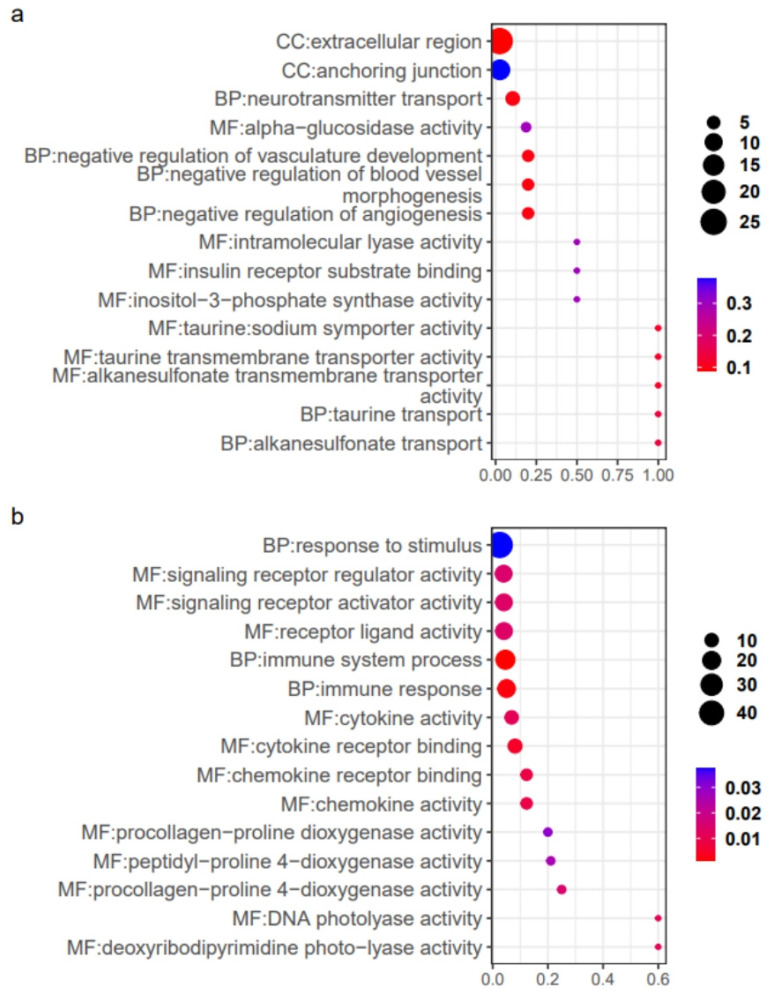
The 15 most significant GO terms for Cluster 2 (**a**) and Cluster 4 (**b**).

Functional enrichment analysis of Cluster 2 identified 312 significant GO terms, with the top 15 pathways including GO:1901343 (negative regulation of vasculature development), GO:0005369 (taurine/sodium symporter activity), and GO:0043560 (insulin receptor substrate binding) (Figure 6a). Notably, four pathways were associated with taurine transmembrane transport, and one was associated with insulin receptor binding. Cluster 4 analysis revealed 537 enriched GO terms, with the top 15 pathways involving signal reception and immune responses, alongside processes such as glucose response (GO:0009749), cytosolic calcium regulation (GO:0051480), and hemoglobin biosynthesis modulation (GO:0046544) (Figure 6b).

### 2.5. Identification of Key Genes for Salt Tolerance

**Figure 7:**
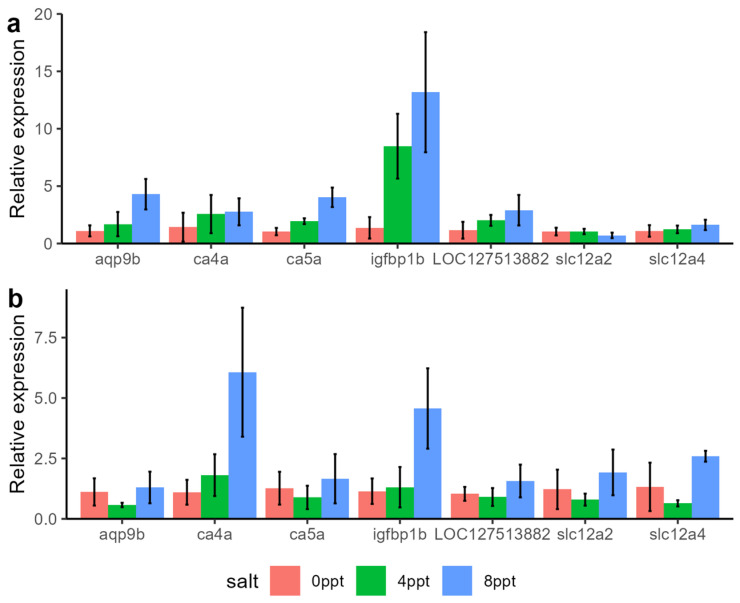
Relative expression levels of key candidate genes. (**a**) The relative expression levels of key candidate genes in gills. (**b**) The relative expression levels of key candidate genes in the intestine.

The genes that show a linear increase in expression levels with increasing salinity may play an important role in the salt tolerance process of grass carp. In this study, based on gene function expression level, we identified seven genes potentially related to salt tolerance in grass carp (Table 1). These genes were highly expressed in the gills or intestines of grass carp in the 8‰ salinity group, and qPCR validation also showed the same trend (Figure 7). Gene functional annotation indicated that these genes not only play an important role in ion transport but also undergo multiple fold changes in expression with increasing salinity.

## 3. Discussion

### 3.1. The Adaptation Range of Carp to Salinity

The salinity of water has a profound impact on the physiology and growth of fish. The salt tolerance of fish varies greatly; marine fish can adapt to higher salinity, e.g., the salinity limitation of brown-marbled grouper (*Epinephelus fuscoguttatus*) is 70 ppt [18] and European bass (*Dicentrarchus labrax*) is 90 ppt [19]. Freshwater fish are less tolerant than marine fish, such as Nile tilapia (*Oreochromis niloticus*), which can tolerate salinity levels of 25–30 ppt [20], and the Chinese sturgeon (*Acipenser sinensis*), after salt domestication, maintained a survival rate of 98.7% in 25 ppt [21]. In grass carp, increasing salinity triggers a series of physiological responses. Numerous studies indicate that salinity levels below 2 ppt have no adverse effects on growth. However, exposure to salinities above 6 ppt induces immune impairment and reduced growth rates. When salinity reaches 11 ppt, feeding cessation occurs, and complete mortality is observed at 17 ppt, confirming 17 ppt as a lethal salinity threshold for this species [22,23,24,25]. Conversely, some studies suggest that moderate salinity elevation may confer protective benefits. For instance, Hong Fang et al. (2022) reported that grass carp exposed to 3 ppt or 6 ppt salinity for 30 days showed no mortality under normal conditions, but following Flavobacterium columnare infection, the mortality rate at 6 ppt was approximately one-third of that observed at 3 ppt [26]. In this study, to investigate the effects of water salinity on grass carp, the experimental fish were acclimated to 8 ppt salinity for 30 days. Compared to other fish species, 8 ppt is considered a relatively low salinity, likely reflecting evolutionary adaptations to their freshwater habitat [27]. In China, most saline–alkaline water bodies exhibit salinity levels of 6–8‰, which significantly impairs grass carp aquaculture. While this salinity range does not drastically increase mortality rates, it severely diminishes economic efficiency due to reduced growth performance and feed conversion. Currently, selective breeding for salt tolerance traits has been successfully implemented in species like tilapia, yielding substantial genetic advancements [28]. These achievements highlight the potential of breeding technologies to develop high-salinity-tolerant grass carp, offering a promising pathway for sustainable aquaculture in saline–alkaline environments.

### 3.2. The Effect of Cortisol on Salt Adaptation of Grass Carp

Cortisol, a critical stress-responsive hormone in fish, is rapidly secreted during stress exposure. In grass carp, high-density aquaculture practices not only induce chronic inflammatory responses but are also associated with elevated cortisol levels; therefore, cortisol is usually used as a stress marker [29]. Salinity, as an environmental stressor, has been extensively documented to interact with cortisol in facilitating fish adaptation to high-salinity environments [29,30,31,32]. Physiologically, cortisol levels typically surge when fish encounter salinity increases during seawater acclimation. For instance, juvenile pink salmon (*Oncorhynchus gorbuscha*) transferred from freshwater to seawater (25‰) exhibited elevated cortisol concentrations within 1 h, a response critical for activating Na^+^/K^+^-ATPase activity, maintaining ion homeostasis, and promoting osmotic regulation [33]. In this study, rising water salinity significantly increased cortisol concentrations in both the gills and intestines, with gill cortisol levels consistently exceeding those in the intestines across all salinity treatments. As a key stress hormone, these cortisol fluctuations confirm that salinity elevation triggered a stress response in grass carp, aligning with prior findings on fish stress physiology.

### 3.3. The Gene Expression Patterns of Gills Under Different Salinity

The gills of freshwater fish play a critical role in osmoregulation and ion exchange, facilitating not only oxygen transport but also the excretion and absorption of sodium, potassium, and chloride ions [34,35]. In this study, transcriptomic sequencing of gill tissues under varying salinity conditions identified 3499 DEGs, significantly exceeding the 1539 DEGs detected in the intestines, indicating that salinity exerts a more profound regulatory effect on gill function. The analysis focused on genes with expression trends correlated to salinity gradients revealed no rapid transcriptional shifts between the 0 ppt and 4 ppt groups, whereas significant expression changes emerged at 8 ppt, suggesting that 4 ppt represents a mild salinity level with negligible physiological disruption in grass carp. GO enrichment analysis of DEGs highlighted distinct functional patterns: upregulated genes in Cluster 1 were predominantly enriched in ion transport pathways, particularly transmembrane regulation of sodium, potassium, and chloride ions, aligning with the previous studies that these ions dominate osmotic pressure regulation in freshwater fish and are actively managed through absorption and excretion mechanisms [36]. Among the downregulated genes, three functional categories predominated. The most prominent group involved small peptide synthesis, primarily comprising the rpl gene family (e.g., *rpl3*, *rpl7*), which encodes ribosomal proteins. The reduced expression implies a potential suppression of non-essential biosynthetic processes under high salinity, allowing for energy conservation for osmoregulation. The second category included hemoglobin-related genes (hbba1, hbba2), critical for systemic oxygen delivery, whose downregulation indicates compromised respiratory function in high-salinity environments. Similar observations in Fat greenling (*Hexagrammos otakii*) and Largemouth bass (*Micropterus salmoides*) [37,38], suggest a conserved adaptive limitation in freshwater fish exposed to saline conditions. The third category encompassed energy metabolism genes, particularly those involved in ATP production and electron transport; this reduced expression may arise from respiratory inhibition, reflecting a systemic energy reallocation to prioritize osmotic balance over metabolic activities.

### 3.4. The Gene Expression Patterns of Intestines Under Different Salinity

The intestine, beyond its role in nutrient absorption, serves as a critical site for water and ion exchange. In this study, functional enrichment analysis of upregulated genes revealed the most significant pathway to be the anchoring junction, involving genes responsible for constructing intercellular connections. Previous studies have identified analogous mechanisms in fish gills, where tight junction proteins act as barriers to restrict paracellular ion leakage [39]. For instance, euryhaline fish transferred to freshwater typically downregulate tight junction proteins to facilitate ion efflux, whereas freshwater fish transitioning to saline environments upregulate these proteins [40]. Here, the observed upregulation of intestinal tight junction genes in grass carp—a novel finding—suggests that intestinal ion transmembrane transport regulation parallels gill mechanisms, potentially restricting ion influx under salinity stress. The second most enriched upregulated pathway, negative regulation of vasculature development, may indicate salinity-induced suppression of intestinal vascular remodeling. The third prominent pathway, taurine transport, is a key osmoregulator in fish [41]. Studies on euryhaline species like the Tongue sole (*Cynoglossus semilaevis*) demonstrate taurine accumulation under both hypo- and hyperosmotic conditions [42], while tilapia transferred to seawater exhibit elevated taurine levels [43]. Dietary taurine supplementation has been shown to mitigate salinity-induced growth suppression and stress in fish [44,45,46], with similar osmoregulatory functions observed in mollusks [47]. The upregulated taurine transporter genes in grass carp likely represent a conserved adaptive mechanism for salinity tolerance. Conversely, downregulated intestinal genes under elevated salinity were predominantly associated with immune-related pathways, a pattern potentially linked to the duration of stress. While acute stress may transiently activate immune responses, chronic stress often suppresses immune function, as documented in previous studies [48].

### 3.5. Identification of Key Genes for Salt Adaptation in Grass Carp

The adaptation of fish to salinity is fundamentally dependent on osmoregulatory mechanisms. While numerous key genes associated with salinity tolerance have been identified across species, critical salinity-responsive genes exhibit species-specific differences. For example, in this study, the widely studied CFTR gene did not show significant differential expression [49,50]. By analyzing expression changes and functional annotations, we identified several genes potentially involved in grass carp salinity adaptation. The first candidate gene is *LOC127513882*, annotated as sodium/potassium-transporting ATPase subunit alpha-1 (Na^+^/K^+^-ATPase α1). In other species, this gene facilitates the exchange of three intracellular Na^+^ ions for two extracellular K^+^ ions, a well-documented mechanism in fish osmoregulation [51]. LOC127513882 in grass carp displayed high baseline expression in the intestine without significant salinity-induced changes, suggesting its primary role in gill-mediated Na^+^/K^+^ transport. The second gene, aquaporin 9b (aqp9b), encodes a water channel protein regulating transmembrane water movement. Aquaporins (AQPs), a family of water-specific transporters, enable water and solute flux under osmotic gradients [52]. Although AQP1 and AQP3 are known to mediate salinity tolerance in fish [53,54], the role of AQP9b remains unclear. Notably, AQP9b mRNA levels increased in *Lateolabrax maculatus* during fresh-water acclimation [55], highlighting its context-dependent regulatory function. Additionally, two carbonic anhydrase (CA) genes were identified. Carbonic anhydrase, a zinc metalloenzyme, plays critical roles in CO_2_ excretion, ion regulation, and acid-base balance by catalyzing the reversible hydration of CO to H^+^ and HCO_3_^−^. In fish, these ions are exchanged for Na^+^ and Cl^−^ across gill epithelia, coupling ion regulation with pH homeostasis [56]. CA4a exhibited pronounced expression changes in both gill and intestine, suggesting its central role in grass carp salinity adaptation, consistent with CA’s osmoregulatory functions in other species [57,58]. The insulin-like growth factor binding protein 1b gene (*igfbp1b*) also emerged as a key gene. Insulin-like growth factors (IGFs), which interact with growth hormone (GH), enhance Na^+^/K^+^-ATPase activity, thereby indirectly supporting salinity adaptation. Studies demonstrate that GH or IGF-I treatment improves salinity tolerance and elevates gill Na^+^/K^+^-ATPase activity [59]. In grass carp, *igfbp1b* expression increased nearly 6-fold in gills at 8 ppt, strongly implicating IGF signaling in salinity adaptation. Finally, two slc12 family genes were identified. The SLC12 family encodes electroneutral cation-coupled chloride cotransporters, essential for cellular volume regulation, neuronal Cl⁻ balance, and epithelial ion transport. *Slc12a2* (nkcc1), a well-characterized osmoregulator, promotes Na^+^/K^+^/Cl^−^ transport, while *slc12a4* (kcc1) mediates K^+^/Cl^−^ flux. Both genes showed salinity-dependent expression changes in gills and intestines, indicating their coordinated roles in maintaining osmotic equilibrium across tissues [11,60].

## 4. Materials and Methods

### 4.1. Experimental Design

In this study, three salinity gradient groups were established: 0 ppt, 4 ppt, and 6 ppt, each containing 40 grass carp with an average weight of 11 ± 1.2 g. All grass carp were kept in plastic containers measuring 0.7 m, 0.5 m, and 0.4 m in length, respectively. Before the experiment, all the fish were acclimated in 0 ppt freshwater for 48 h, after which the salinity was gradually increased by 1 ppt every 12 h until the specified concentration was reached. The experiment began once the 8 ppt group reached the target salinity and lasted for 30 days. During the experiment, all fish were fed the same diet twice daily (at 08:00 and 19:00). Half of the water was replaced with water of the same salinity every two days. After 30 days, the grass carp were fasted for 12 h prior to sampling. Before sampling began, all fish were euthanized using MS-222, and then the intestine and second-gill arch were sampled for transcriptome sequencing and cortisol concentration detection.

### 4.2. Cortisol Concentration Detection

Utilize the Fish Cortisol ELISA kit to measure cortisol levels in the intestine and gills. The detection method is as follows: (1) Weigh 0.1 g of tissue, add physiological saline, and homogenize. Centrifuge at 3000 rpm for 10 min and then collect the supernatant for subsequent steps. (2) Prepare 6 blank wells, 6 standard wells, and 30 sample wells. The blank wells will remain untreated. Add 50 μL of varying concentrations of standards to the standard wells. For the sample wells, add 10 μL of the supernatant followed by 40 μL of sample diluent; (3) Add 100 μL of horseradish peroxidase (HRP)-labeled detection antibody to both the standard and sample wells. Seal the reaction wells and incubate at 37 °C for 60 min; (4) Remove the liquid, add a washing buffer, and let it sit for 1 min. Remove the washing buffer and repeat this step 5 times. (5) Add 50 μL of substrates A and B and incubate at 37 °C in the dark for 15 min. (6) Add 50 μL of termination solution and measure the optical density (OD) at a wavelength of 450 nm. (7) Subtract the average absorbance of the blank wells from the OD values of the standard and sample wells to obtain the true absorbance. Then, fit a linear equation using the true absorbance values of the standard wells. Input the sample absorbance into this equation to determine the true concentration of cortisol in the different samples.

### 4.3. RNA Extraction and Transcriptome Sequencing

The MJZol total RNA extraction kit was used to extract total RNA from tissue samples. The concentration and purity of the extracted RNA were assessed using a Nanodrop2000 (Thermo Fisher Scientific, Waltham, MA, USA). Agarose gel electrophoresis was employed to evaluate RNA integrity, and the RQN value was determined using an Agilent 5300 Fragment Analyzer (Agilent Technologies, Santa Clara, CA, USA). Only samples with a total RNA of at least 1 µg, a concentration of ≥30 ng/μL, an RQN > 6.5, and an OD260/280 ratio between 1.8 and 2.2 were used for sequencing. The RNA library was constructed using the Illumina^®^ Stranded mRNA Prep (Illumina, San Diego, CA, USA), Ligation Method. Then, the library was sequenced on the NovaSeq X Plus platform (Illumina, San Diego, CA, USA).

### 4.4. Data Analysis

For raw sequencing data, quality control was performed using fastp (v0.23.4) [61], which included the following steps: (1) Removing adapter sequences; (2) Trimming low-quality bases; (3) Removing reads with an “N” ratio exceeding 10%; and (4) Removing reads shorter than 20 bp. After quality control, the clean data were mapped to the grass carp reference genome using HiSat2 software (v2.2.1) [62], and then StringTie (v2.2.1) was employed to assemble the transcripts [63]. TransDecoder (v5.5.0) was utilized for redundancy removal and protein sequence prediction. Subsequently, the predicted protein sequences were aligned to the Nr, Gene ontology (GO) databases using blastp software (v2.9.0). DEGs were identified using DESeq2 (v1.42.0), with the following criteria [64]: FDR < 0.05 and |log2FC| ≥ 1. Enrichment analysis was conducted using goatools (v1.4.4) [65].

### 4.5. qPCR Validation

Total RNA was extracted using the TRIzol reagent kit following the manufacturer’s protocol. RNA integrity and concentration were assessed by 1% agarose gel electrophoresis and measured using a Cytation 5 multimode microplate reader (Agilent Technologies, CA, USA). First-strand cDNA was synthesized using the PrimeScript™ RT Reagent Kit (Takara Bio, Shiga, Japan) with gDNA Eraser. Quantitative real-time PCR (qRT-PCR) was performed on a 96 Real-Time PCR System (Roche Diagnostics, Penzberg, Germany). The cDNA templates were diluted 5-fold for qRT-PCR reactions. The thermal cycling conditions were as follows: 95 °C for 30 s (initial denaturation); 40 cycles of 94 °C for 5 s (denaturation), 60 °C for 30 s (annealing), and 72 °C for 30 s (extension); followed by a melting curve analysis at 95 °C for 5 s, 60 °C for 1 min, and 95 °C for 1 s. The β-actin gene was used as an internal reference, and relative gene expression levels were calculated using the 2^−ΔΔCt^ method. Primer sequences for the target genes are listed in Table 2, and all primers were synthesized by Sangon Biotech (Shanghai, China).

## 5. Conclusions

In this study, cortisol levels measured after 30 days of salinity acclimation were strongly associated with salinity tolerance in grass carp. Transcriptomic sequencing of gill and intestinal tissues under varying salinity conditions indicated that ion exchange processes primarily occurred in the gills, while the intestine showed significant upregulation of taurine transporter-related genes, suggesting a potential role for taurine in osmotic regulation. Additionally, based on expression patterns and functional annotations, seven genes were identified as likely contributors to salinity adaptation in grass carp: *LOC127513882* (Na^+^/K^+^-ATPase α1), *aqp9b* (aquaporin 9b), *ca4a* (carbonic anhy-drase 4a), *igfbp1b* (insulin-like growth factor binding protein 1b), *slc12a2* (*nkcc1*), *slc12a4* (*kcc1*).

## Figures and Tables

**Table 1:** Candidate key genes for salt adaptation in grass carp.

Gene Name	Gene Description	Gill ppt0	Gill ppt4	Gill ppt8	Intestine ppt0	Intestine ppt4	Intestine ppt8
*LOC127513882*	sodium/potassium-transporting ATPase subunit alpha-1	20.68	18.66	63.82	103.44	219.61	174.92
*aqp9b*	aquaporin 9b	8.54	9.44	30.48	0.19	0.07	0.21
*ca4a*	carbonic anhydrase IV a	1.01	0.46	23.79	1.90	3.34	6.93
*ca5a*	carbonic anhydrase Va	21.88	59.77	86.33	43.72	58.34	49.63
*igfbp1b*	insulin-like growth factor binding protein 1b	207.49	204.60	1250.15	4.94	11.53	35.71
*slc12a2*	solute carrier family 12 member 2	0.47	0.92	1.09	3.03	2.96	5.56
*slc12a4*	solute carrier family 12 member 4	2.98	2.35	5.53	1.86	1.61	3.31

**Table 2:** The qPCR primer of the 7 key candidate genes.

Primer	Sequences (5′–3′)
β-Actin-RT-F	GATGATGAAATTGCCGCACTG
β-Actin-RT-R	ACCGACCATGACGCCCTGATGT
LOC127513882-RT-F	GGGTGCCATTGTAGCCGTAA
LOC127513882-RT-R	CCATGTCCGTTCCCAGGTC
aqp9b-RT-F	CATCCACTTTGGCTTTACTC
aqp9b-RT-R	TGTGGCATTTACACCAGTTA
ca4a-RT-F	AAAGCACCATAAAGAACAACG
ca4a-RT-R	CCTCATAGAAGAATCCCAACA
ca5a-RT-F	TATTGACATCGTGGTGCGTAA
ca5a-RT-R	TCCTCCAGTGGTCCTCCCT
igfbp1b-RT-F	ACAGCAGATGTTAGGCGAGAAG
igfbp1b-RT-R	CAGCCGATAAATCATCAGTTCC
slc12a2-RT-F	TGCTGGACTGGGTAGATTGA
slc12a2-RT-F	GGAGGAGGGTTTGGATGA
slc12a4-RT-F	GTGCCCAAGTCACCGAATA
slc12a4-RT-F	GCGAAAGTTGTGCCTAAATAA

## Data Availability

The transcriptome sequencing data are available in the NCBI SRA database under the BioProject access code PRJNA1219338.

## Supplementary Materials

The following supporting information can be downloaded at https://www.mdpi.com/article/10.3390/ijms26072930/s1.

## Abbreviations

DEGs	Differentially expressed genes
AQPs	Aquaporins
qRT-PCR	Quantitative real-time PCR
