# Supplementary figures and images for: RNA-Seq Reveals Adaptation Strategy in Grass Carp (Ctenopharyngodon idella) Under Hypersaline Conditions
Source: Int J Mol Sci. 2025 Mar 24;26(7):2930. doi: 10.3390/ijms26072930 (PMC11989157; doi:10.3390/ijms26072930)

# Optimal number of clusters

Elbow method

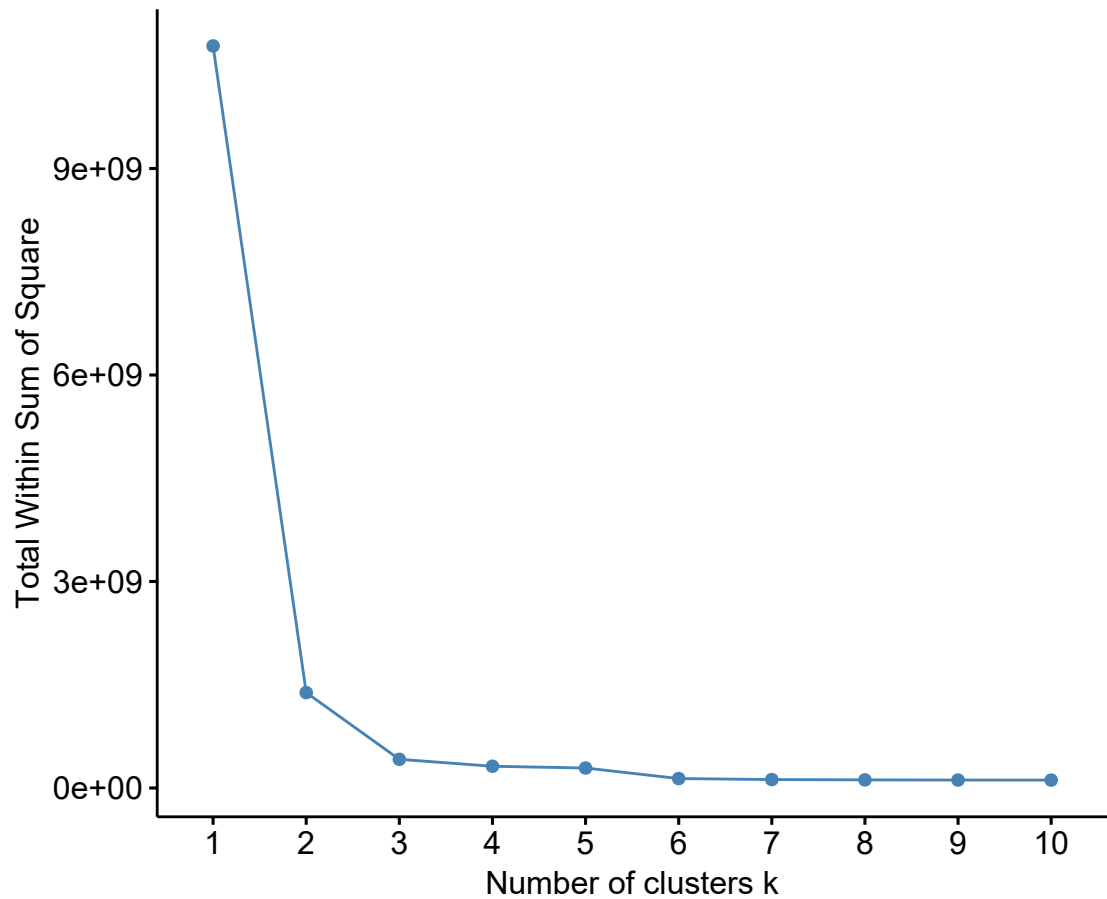

Supplement: Supplementary file 1 [file ijms-26-02930-s001.zip › FigureS2.pdf]

# Optimal number of clusters

Elbow method

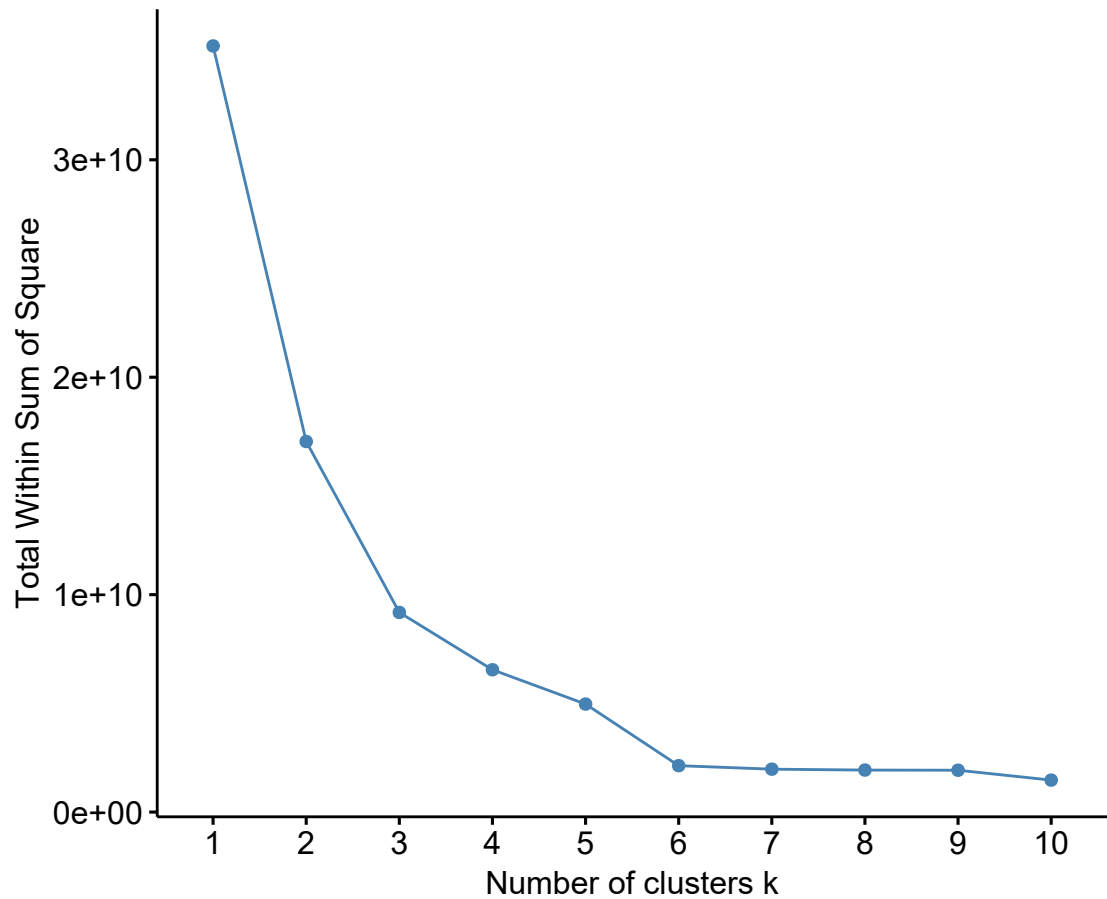

Supplement: Supplementary file 1 [file ijms-26-02930-s001.zip › FigureS1.pdf]
